# Heat exposure impairs porcine oocyte quality with suppressed actin expression in cumulus cells and disrupted F-actin formation in transzonal projections

**DOI:** 10.1186/s40104-020-00477-8

**Published:** 2020-07-06

**Authors:** Chao Yin, Jie Liu, Zhanglin Chang, Bin He, Yang Yang, Ruqian Zhao

**Affiliations:** 1 MOE Joint International Research Laboratory of Animal Health & Food Safety, Nanjing Agricultural University, Nanjing, Jiangsu China; 2 Key Laboratory of Animal Physiology and Biochemistry, Ministry of Agriculture, Nanjing Agricultural University, No.1 Weigang Road, Nanjing, 210095 China; 3 College of Animal Science and Technology, Jiangxi Agricultural University, Nanchang, Jiangxi China

**Keywords:** Actin, Cumulus cell, Gilt, Heat stress, Oocyte

## Abstract

**Background:**

Transzonal projections (TZPs) constitute a structural basis for the communication between the oocyte and its surrounding cumulus cells (CCs), which play critical roles in promoting the oocyte maturation. Previously we found that heat stress (HS) causes loss of TZPs in porcine cumulus-oocyte complexes (COCs) with decreased density of filamentous actin (F-actin). However, the time-course responses of F-actin and its monomeric actins (β-actin and γ-actin) during the *in vitro* maturation of oocytes remain unclear.

**Results:**

In this study, excised porcine ovaries were exposed to HS at 41.5 °C for 1 h before COCs were isolated and matured *in vitro* for 44 h. HS significantly reduced oocyte quality, characterized by impaired cumulus expansion, delayed meiotic resumption and lower survival rate and polar body extrusion rate, as well as decreased expression of mitochondrial DNA-encoded genes and elevated mitochondrial reactive oxygen species concentration. Expression of β-actin and γ-actin in CCs increased gradually with oocytes maturation, which was significantly reduced in HS group, especially at 24 h and/or 44 h of *in vitro* maturation. By contrast, the number of TZPs and the fluorescence intensity of F-actin in zona pellucida decreased gradually during oocytes maturation, which were significantly reduced by HS at 24 h of *in vitro* maturation. Moreover, colocalization analyses revealed both β-actin and γ-actin contribute to the F-actin formation in porcine TZPs, and the colocalization of F-actin with GJ protein connexin 45 was significantly reduced in heat-exposed COCs.

**Conclusions:**

The results indicate that the suppression of actin expressions in CCs, which may lead to the F-actin unstabilization in TZPs, will subsequently contribute to the compromised quality of oocytes under HS.

## Background

In mammals, oocytes lose contacts with the external environment once they enter the antral follicle stages. Instead, their growing demands of materials are maintained by the surrounding cumulus cells (CCs), which form the cumulus-oocyte complexes (COCs) with oocytes. Transzonal projections (TZPs) are one class of intercellular structures linking CCs with oocytes. They extend from the innermost layers of CCs and penetrate the intervening zona pellucida to contact oocyte with gap junction (GJ) structures distributed on the oolemma [1]. Through TZPs, CCs provide oocytes with multifarious nutrients and signaling molecules, such as ions, hormones, second messengers, small metabolic intermediates [2], and even mRNA and lipids [3, 4].

TZP is mainly constituted by the filamentous actins (F-actin) [5], one cluster of toruliform structures polymerized by the activated monomeric actins (G-actin), including β-actin and γ-actin [6, 7]. Previous studies have demonstrated that the number and functional status of TZPs would change dynamically according to the developmental stages of the oocytes. For example, in humans, TZPs structures are particularly numerous at the pre-antral stages of oocytes, while during the following antral stages their number and complexity of contact at the oocyte surface decrease gradually with the oocyte development [8]. However, in pigs, the time-course responses of F-actin and its monomeric actins (β-actin and γ-actin) during the oocyte *in vitro* maturation remain unclear.

Seasonal hyperthermia-induced fertility reductions, such as the declined conception rate and the depressed embryonic development potentials, were widely observed in female animals including pigs [9], cows [10] and mouse [11]. The primary cause is the environmental heat stress (HS)-induced quality reductions in oocytes [12–15], of which the mechanisms refer to the mitochondrial dysfunctions, oxidative stress and the cell apoptosis, in both oocytes [12, 16, 17] and granulosa cells [18, 19]. Meanwhile, we previously found that poor oocyte quality in heat-stressed porcine COCs is also related to the TZPs disruptions [20]. Similarly, Yin et al. [21] demonstrated that HS increases the apoptosis through F-actin aggregation in mouse H9C2 cardiomyocytes. Guo et al. [22] reported that moderate hyperthermia exposure at 39 °C significantly increases the alpha 1 actin gene expression in C2C12 cells, thus accelerates the growth of sarcomeres in myofibrils. These findings suggest that HS may also cause TZPs dysfunctions through the disruptions of G-actins expression or the F-actin organizations. However, it is still undefined whether G-actin expression and F-actin formation are altered by HS during the porcine COCs *in vitro* maturation.

Therefore, to provide a dynamic profile of F-actin organization and the expression of monomeric G-actins during porcine oocyte maturation and to reveal the effects of HS on TZPs disruption, structure of TZPs and the expression of β-actin and γ-actin were investigated during the COCs *in vitro* maturation by using an ovarian heat stress model. The results will provide new insights into the underlying mechanisms of oocyte quality impairments induced by HS.

## Materials and methods

### Cell isolation and oocyte *in vitro* maturation

Ovaries were dissected from cross-bred prepubertal gilts (Landrace × Large White × Duroc; 135 to 170 days of age; 70 to 120 kg of body weight) slaughtered in a local abattoir. Approximately one hundred ovaries in the follicular phase of the ovarian cycle were selected and kept in 0.9% saline (w/v) supplemented with 75 μg/mL potassium penicillin G and 50 μg/mL streptomycin sulfate at 37 °C and transported to the laboratory within 3 h. Ovaries were equally assigned to control group (Control) and heat stress group (Heat) at random, and then transferred into 38.5 °C and 41.5 °C water bath for 1 hour, respectively, according to the previous publication by Pennarossa, et al. [23]. Afterwards, 400 to 500 COCs from each group were aspirated from ovarian follicles of 3 to 6 mm in size with an 18-gauge needle connected to a 20-mL disposable syringe. Only the COCs surrounded by at least five layers of compact cumulus cells and evenly granulated ooplasm were selected for subsequent culture. After washing three times in HEPES-buffered tissue culture medium 199 (TCM-199) plus 0.8 mmol/L *L*-glutamine and 2% (v/v) bovine serum, COCs were cultured in groups of 80 to 100 in 4-well dishes (*n* = 4) with TCM-199 supplemented with 10% (v/v) bovine serum, 10% (v/v) porcine follicular fluid, 0.8 mmol/L *L*-glutamine, 75 μg/mL potassium penicillin G, 50 μg/mL streptomycin sulfate, 15 IU/mL pregnant mare’s serum gonadotropin and 15 IU/mL human chorionic gonadotropin at 38.5 °C with 5% CO_2_, 20% O_2_ and maximum humidity for another 24 h or 44 h, as described earlier [20]. Finally, viable oocytes and their companion cumulus cells were collected at different stages of *in vitro* maturation (0 h, 24 h and 44 h) for further analyses.

### Evaluation of cumulus expansion and cell viability

Cumulus expansion was measured during the COCs *in vitro* maturation period, as described previously [24]. Briefly, at 8 h, 16 h, 24 h and 44 h of *in vitro* maturation, 30 COCs from each experimental group were taken out of the incubators, respectively, to capture the digital images with a charge coupled device (CCD) camera. The size of each COC was measured from digital images. The total two-dimensional area of each COC was expressed as the total number of pixels using the threshold and measure functions of ImageJ version 1.50i National Institutes of Health, USA [25]. Relative cumulus expansion levels were calculated for each COC, among which the value at 0 h was considered as the basis for comparison, with a value of 1. Afterwards, these COCs were digested with hyaluronidase (Hya), and the separated CCs and oocytes were used to assess their survival rates under the light microscope (Leica S8AP0). Briefly, CCs were stained with 0.2% trypan blue solution immediately after the Hya digestion. The numbers of dead and viable cells were assessed using manual cells counts under the light microscope based on the development of blue color. Simultaneously, oocytes were defined as having morphologically survived if they possessed an intact zona pellucida and plasma membrane, translucent appearance of cytoplasm, normal size of the perivitelline space, and extruded polar body. The viabilities of both oocytes and CCs were calculated as the percentage of viable cells out of total cells number. The experiments of cumulus expansion and cell viability evaluation were performed in triplicate.

### Analysis of mitochondrial distribution

Thirty oocytes from each experimental group were stained with 200 nmol/L Mito-Tracker Red FM (Molecular Probes, USA, Cat# M22425) for 30 min at 38.5 °C under 5% CO_2_, followed by washing in TCM-199 for three times (5 min each). Then the oocytes were mounted on glass slides and observed with a laser-scanning confocal microscope (Zeiss LSM 710 META, Germany). The parameters including the laser intensity, exposure time and contrast were set during the image acquisition period of the first oocyte, which would never be changed during the experiment. The images were captured at the equatorial plate of each oocyte, where the oocyte has a maximum diameter, to keep the consistency of the experimental conditions.

### Determination of mtDNA copy number

Eighty oocytes or their companion CCs were pooled as one sample (four samples per group) to extract the total DNA. Briefly, the pooled oocytes and CCs were incubated in a lysis solution containing 0.5 mol/L of EDTA, pH 8.0 and 2 mg/mL of proteinase K (Amresco, USA) at 37 °C for 50 min. The specific primers for the coding region of mitochondrial DNA were used for the quantification of mtDNA copy number, and primer specific for the nuclear-encoded reference gene PPIA was used for standardization (Table 1). The relative mtDNA copy number was determined by real-time PCR as previously described and then calculated with 2^−^^△△Ct^ method [20].

**Table 1:** Primer sequences for realtime PCR

Target genes	Primer sequences (5′ to 3′)	Products, bp	GenBank No.
18S	F: CCCACGGAATCGAGAAAGAG R: TTGACGGAAGGGCACCA	122	AY265350.1
*PPIA*	F: GACTGAGTGGTTGGATGG R: TGATCTTCTTGCTGGTCTT	116	NM_214353.1
*β-actin*	F: CATCACCATCGGCAACGA R: GCGTAGAGGTCCTTCCTGATGT	144	XM_021086047.1
*γ-actin*	F: TCAGCAAGCAGGAGTACGAC R: GAGGTGTGTACATTTGCCAGG	140	XM_003357928.4
*COX1*	F: TGGTGCCTGAGCAGGAATAGTG R: ATCATCGCCAAGTAGGGTTCCG	88	KF888634.1
*COX2*	F: GCTTCCAAGACGCCACTTCAC R: TGGGCATCCATTGTGCTAGTGT	154	KF888634.1
*COX3*	F: GGCTACAGGGTTTCACGGGTTG R: TCAGTATCAGGCTGCGGCTTCA	130	KF888634.1
*ND1*	F: TCCTACTGGCCGTAGCATTCCT R: TTGAGGATGTGGCTGGTCGTAG	165	KF888634.1
*ND2*	F: ATCGGAGGGTGAGGAGGGCTAA R: GTTGTGGTTGCTGAGCTGTGGA	191	KF888634.1
*ND3*	F: AGCACGCCTCCCATTCTCAAT R: TGCTAGGCTTGCTGCTAGTAGG	172	KF888634.1
*ND4*	F: TCGCCTATTCATCAGTAAGTCA R: GGATTATGGTTCGGCTGTGTA	174	KF888634.1
*ND5*	F: CGGATGAGAAGGCGTAGGAA R: GCGGTTGTATAGGATTGCTTGT	103	KF888634.1
*ND6*	F: ACTGCTATGGCTACTGAGATGT R: CTTCCTCTTCCTTCAACGCATA	124	KF888634.1
*ND4L*	F: GATCGCCCTTGCAGGGTTACTT R: CTAGTGCAGCTTCGCAGGCT	182	KF888634.1
*ATP6*	F: ACTCATTCACACCCACCACACA R: CCTGCTGTAATGTTGGCTGTCA	232	KF888634.1
*ATP8*	F: TGCCACAACTAGATACATCC R: GCTTGCTGGGTATGAGTAG	107	KF888634.1
*CYTB*	F: CTGAGGAGCTACGGTCATCACA R: GCTGCGAGGGCGGTAATGAT	162	KF888634.1

### RNA extraction, reverse transcription and real-time quantitative PCR

Thirty oocytes or their companion CCs were pooled as one sample (four samples per group). The total RNA extraction and reverse transcription were conducted using the SuperScriptTM III First-Strand Synthesis System (Invitrogen, USA) according to the manufacturer’s protocol. The total RNA extracted from CCs was quantified with the NanoDrop ND-2000 Spectrophotometer (ThermoFisher, USA) and the same quantity (800 ng) of total RNA was used in the reverse transcription. Subsequently, four microliters of diluted cDNA (1:10, v/v) was used for quantitative real-time PCR, which was performed with Mx3000P (Stratagene, USA). 18S rRNA and PPIA were chosen as reference genes in CCs to normalize the technical variations, and the oocyte number was employed to calibrate the target genes’ expression levels in oocytes. All primers were synthesized by Generay (Shanghai, China) (Table 1). The real-time PCR results were analyzed using the 2^−△△Ct^ method, and the abundance of mRNA was expressed as the fold change relative to the mean value of the control group.

### Protein extraction and Western blot analysis

For protein extraction, 1 hundred oocytes or their companion CCs were pooled as one sample (four samples per group) and lysed in Laemmli sample buffer (SDS sample buffer with 2-mercaptoethanol), and boiled at 100 °C for 5 min. Approximately 15 μg of total protein from each sample were subjected to 10% SDS-polyacrylamide gel electrophoresis (PAGE). Mouse anti-β-actin antibody (AP0060, Bioworld, 1:200) and mouse anti-γ-actin antibody (ab123034, Abcam, 1:200) were used as primary antibodies. Western blot analyses were carried out according to the recommended protocols provided by the manufacturers. Coomassie brilliant blue staining method was used to calibrate the target proteins expression levels, as reported previously [26]. Images were captured by VersaDoc 4000MP system (Bio-Rad, Hercules, CA) and the band density was analyzed with Quantity One software (Bio-Rad).

### Evaluation of mROS content in CCs and oocytes

Fluorescent dye MitoSOX Red (Invitrogen, USA, Cat# M36008) were used to analyze the mROS generation in CCs and oocytes. Briefly, CCs isolated from the same well of COCs were pooled as one sample (four samples per group), and stock solutions of MitoSOX Red were diluted in PBS and added to the isolated CCs at 1 × 10^6^ cells/mL to give final concentrations of 2 mmol/L and incubated for 30 min at 37 °C under 5% CO_2_, followed by washing in TCM-199 twice. Samples were then centrifuged for 5 min at 600×*g*, and the pellets were resuspended in 0.5 mL PBS and subsequently transferred to 5 mL FACS tubes. The MitoSOX Red fluorescence was then measured on a flow cytometer, according to the previous study [27]. For oocyte mROS analysis, 30 denuded oocytes from each group were incubated in MitoSOX Red (10 μmol/L in PBS) at 38.5 °C, 5% CO_2_ for 30 min followed by washing twice in PBS. Stained oocytes were transferred and mounted on glass slides, and then observed with a laser-scanning confocal microscope (Zeiss LSM 710 META, Germany). Afterwards, the captured pictures were converted into 8-bit grayscale maps in ImageJ software. After selecting the target area of ooplasm with an oval selections tool, the grayscale value of each target area was measured, which represented the fluorescence intensity of MitoSOX Red in the oocyte.

### Immunofluorescence (IF) and F-actin staining

Briefly, 30 oocytes from each group were fixed in 4% paraformaldehyde for 15 to 20 min at room temperature. Oocytes were then transferred to a membrane permeabilization solution (1% Triton X-100 in PBS) for 8 to 12 h at 4 °C. After 1 h in blocking buffer (1% bovine serum albumin in PBS), oocytes were subsequently incubated overnight at 4 °C with rabbit anti-connexin 45 antibody (marker protein of GJs, BS3470, Bioworld, 1:100), mouse anti-β-actin antibody (AP0060, Bioworld, 1:200), mouse anti-γ-actin antibody (ab123034, Abcam, 1:200) or rabbit anti-tubulin α (ab52866, Abcam, 1:250) antibody. After three washes (5 min each) in wash buffer (0.1% Tween 20 and 0.01% Triton X-100 in PBS), oocytes were incubated with Alexa Fluor 488 (Molecular Probes, A11008, 1:500) or Alexa Fluor Plus 555 (Molecular Probes, A32727, 1:1000) secondary antibody for 1 h at 38.5 °C under 5% CO_2_. For nuclear and F-actin staining, oocytes were sequentially washed for three times (5 min each) after the blocking or secondary antibody incubation operations, and transferred into the staining solution containing the fluorescent dye Hoechst 33342 (Invitrogen, USA, Cat# H3570) or TRITC Phalloidin (Yeasenbio Co., Ltd., China, Cat# 40734ES75) at a final concentration of 10 μmol/L or 200 nmol/L, respectively, and then incubated for another 15 to 30 min at 38.5 °C under 5% CO_2_. Finally, after three washes in wash buffer, oocytes were mounted on glass slides, and examined with a confocal laser-scanning microscope (Zeiss LSM 700 META, Germany). TZPs numbers and the colocalization of TZPs and monomeric actins in the zona pellucida areas were calculated manually. The fluorescence intensity of F-actin co-localized with connexin 45 in oolemma areas were quantified by ImageJ software.

### Statistical analysis

All data are presented as mean ± SEM. Descriptive statistics was performed to check the normality and homogeneity of variances before the parametric analyses, and log_10_ transformation was performed before statistical analysis when the data distribution was not normal. Differences between two or multiple groups were analyzed by using independent samples t-test and one-way ANOVA with SPSS 18.0 software (SPSS Inc., Chicago, IL, USA) for windows, respectively. ANOVA followed by *post hoc* Dunnett test was used to determine statistical differences between groups and Chi-square test was used to evaluate the difference in mitochondrial distribution between two groups. The method of 2^−ΔΔCt^ was used to analyze the real-time PCR data. The differences were considered statistically significant when *P* < 0.05.

## Results

### Establishment of porcine ovarian HS model

**Fig. 1:**
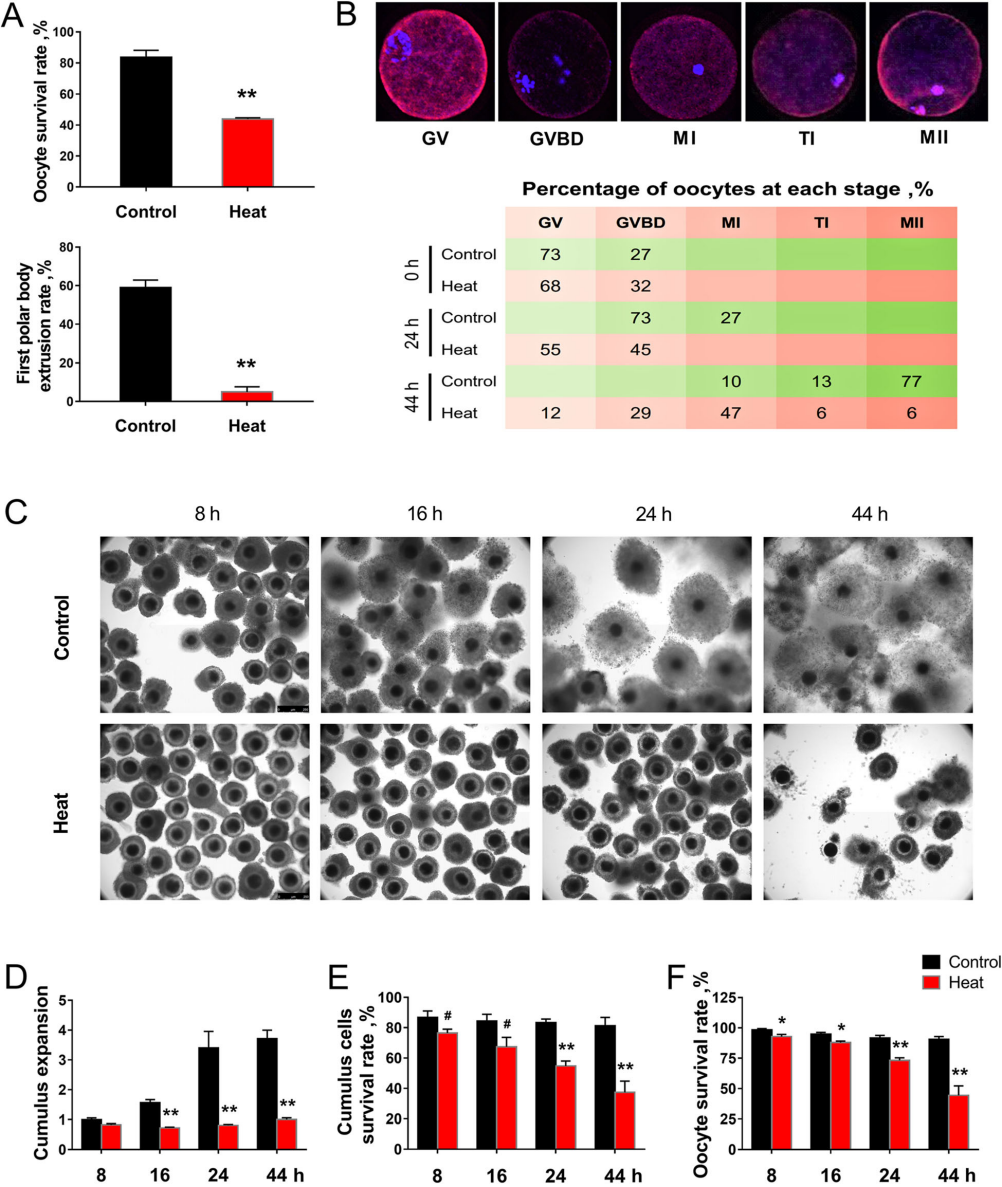
Oocyte quality and cumulus expansion analysis under HS. (**a**) Oocyte survival rate and the first polar body extrusion rate after 1 h of ovarian HS (*n* = 4); (**b**) Evaluation of oocyte maturational progression after HS exposure (*n* = 30). (**c**-**f**) Representative images (magnification, × 10) and the statistical analysis of cumulus expansion patterns (**c**-**d**) and the survival rates of oocytes and CCs (**e**-**f**) at different stages during *in vitro* maturation after 1 h of 41.5 °C HS (*n* = 4). GV: Germinal vesicle; GVBD: Germinal vesicle break down; MI: First meiotic metaphase; TI: First meiotic telophase; MII: Second meiotic metaphase. Data are expressed as mean ± SEM. Control, ovaries were kept in 38.5 °C water bath for 1 h, and then the isolated COCs were cultured in 38.5 °C for another 8 h, 16 h, 24 h or 44 h; Heat, ovaries were kept in 41.5 °C water bath for 1 h, and then COCs were isolated and cultured in 38.5 °C for another 8 h, 16 h, 24 h or 44 h. *P < 0.05, **P < 0.01 and ^#^ 0.05 < *P* < 0.1, compared with control; a, b, c, mean values not sharing the same letters are significantly different, *P* < 0.05; ND, no detected

The survival rate, the first polar body extrusion rate and the meiotic progression were evaluated to investigate the effect of HS on porcine oocyte maturation. Ovaries heat-stressed for 1 hour significantly decreased (*P* < 0.05) the survival rate and the first polar body extrusion rate of oocytes after 44 h of *in vitro* maturation (Fig. 1a). The meiotic progression of oocytes was delayed by HS, with 55% of the oocytes lag behind at the stage from GV to GVBD at 24 h of *in vitro* maturation, and only 6% of the oocytes reached MII stage in HS group at 44 h compared to 77% in control group (Fig. 1b).

Secondly, COCs were isolated and cultured for 8 h, 16 h, 24 h and 44 h *in vitro* after 1 h of ovarian HS to evaluate the influence of *in vitro* maturation durations on the CCs and oocyte functions. Results showed that the amount and the number of layers of CCs surrounding the control group oocytes increased gradually during the COCs *in vitro* maturation, whereas in HS group the COCs were not adequately expanded, characterized by remarkable decreases (*P* < 0.05) in cumulus expansions at 16 h, 24 h and 44 h of COCs *in vitro* maturation, as compared with control groups (Fig. 1c, d). Moreover, ovarian HS observably reduced (*P* < 0.05) the survival rates of both CCs and oocytes at 24 h, 44 h and 8 h, 16 h, 24 h, 44 h of *in vitro* maturation, respectively (Fig. 1e, f).

### Ovarian HS reduces mitochondrial functions in CCs and oocytes

**Fig. 2:**
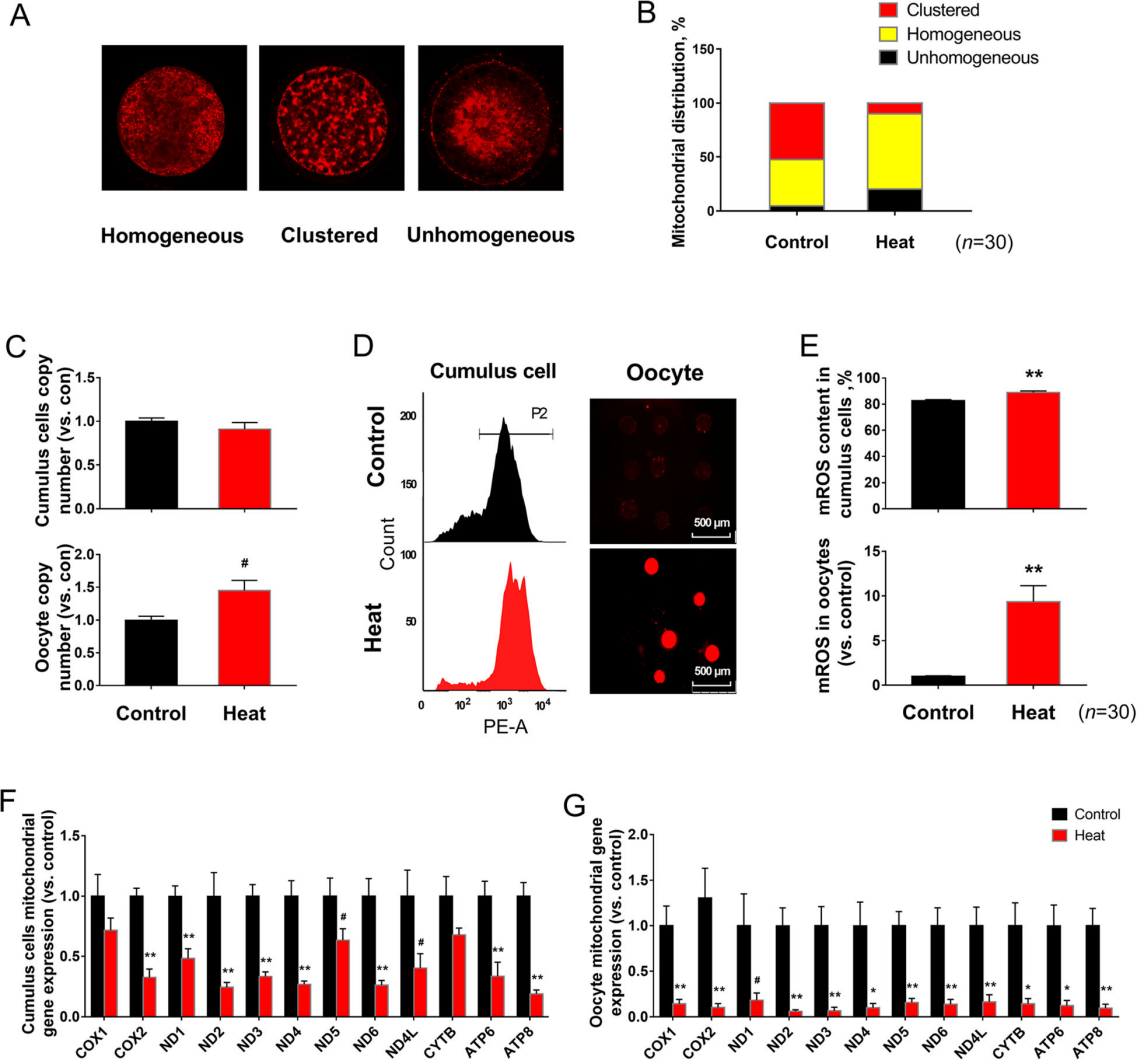
Mitochondrial functions analysis in CCs and oocytes post heat stress. (**a**-**b**) Representative images and the ratios of different mitochondrial distribution patterns in oocytes; (**c**) mtDNA copy number of CCs and oocytes (*n* = 4); (**d**-**e**) Representative images and the content of mROS in CCs and oocytes (*n* = 30); (**f**-**g**) mtDNA-encoded genes expression levels in CCs and oocytes (*n* = 4). Data are expressed as mean ± SEM. Control, ovaries were kept in 38.5 °C water bath for 1 h, and then the isolated COCs were cultured in 38.5 °C for 24 h; Heat, ovaries were kept in 41.5 °C water bath for 1 h, and then COCs were isolated and cultured in 38.5 °C for another 24 h. *P < 0.05, **P < 0.01 and # 0.05 < *P* < 0.1, compared with control

Three different patterns of mitochondrial distribution in oocytes were detected with fluorescent dye Mito-Tracker Red, including the homogeneous distribution, the clustered distribution and the unhomogeneous distribution (Fig. 2a). The percentage of clustered distribution was significantly lower (*P* < 0.05), whereas the percentage of homogeneous distribution was significantly higher (*P* < 0.05) in HS-treated oocytes than in control groups. No significant difference was observed in the percentage of inhomogeneous distribution between control and HS group (Fig. 2b).

No change was observed in mtDNA copy number in both CCs and oocytes between groups (Fig. 2c), while mROS in CCs and oocytes from HS group were more concentrated (*P* < 0.05) than those of control group (Fig. 2d, e). At the same time, the expression levels of mtDNA-encoded genes were significantly down-regulated (*P* < 0.05) in HS group CCs and oocytes, when compared with control group (Fig. 2f, g).

### G-actins gene and protein expressions in CCs during the oocyte development

**Fig. 3:**
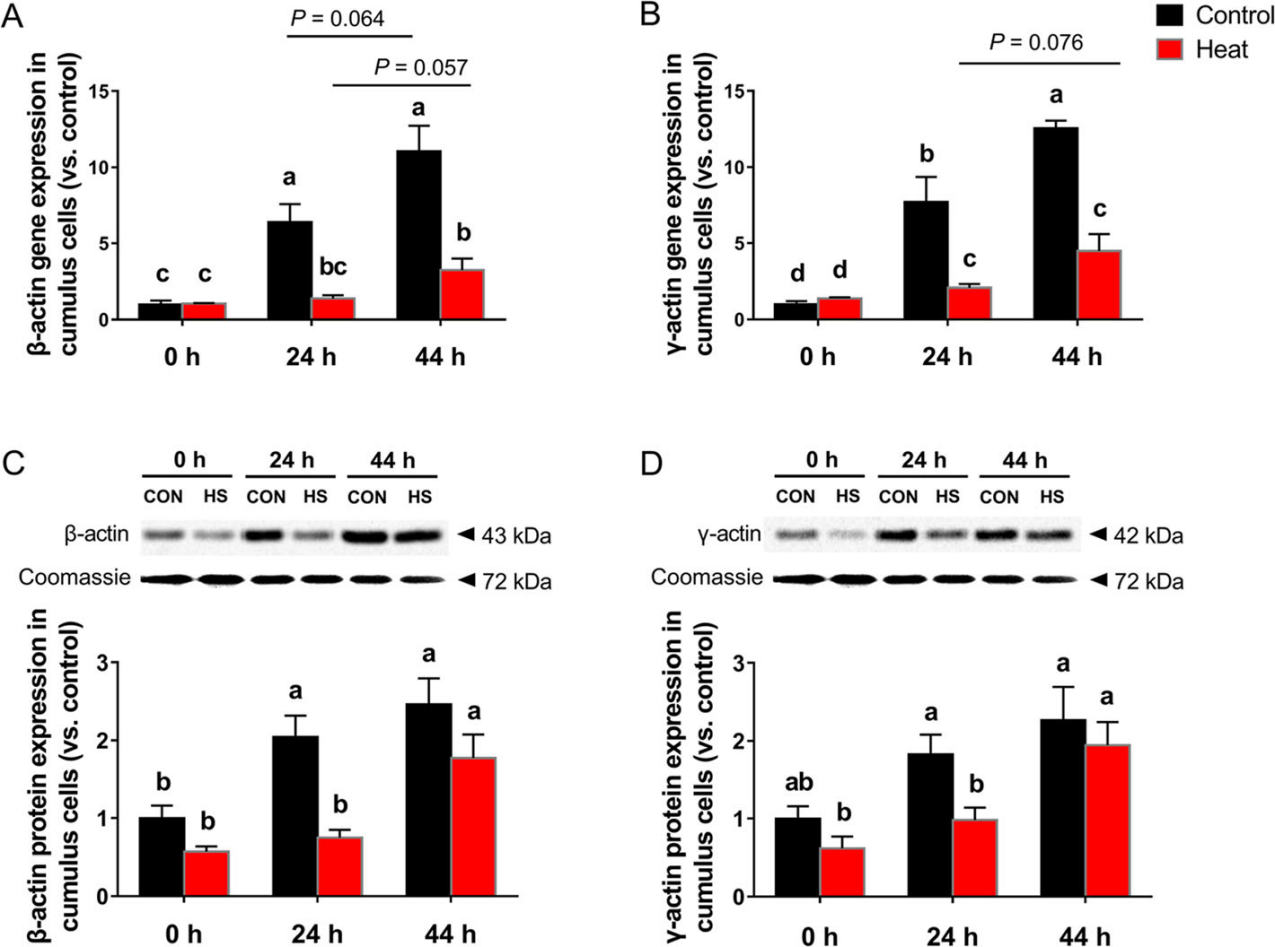
G-actins gene and protein expressions in CCs after heat exposure. (**a**-**b**) Gene expression levels of β-actin and γ-actin in CCs; (**c**-**d**) Protein expression levels of β-actin and γ-actin in CCs. Data are expressed as mean ± SEM. Control, ovaries were kept in 38.5 °C water bath for 1 h, and then the isolated COCs were cultured in 38.5 °C for 0, 24 or 44 h; Heat, ovaries were kept in 41.5 °C water bath for 1 h, and then COCs were isolated and cultured in 38.5 °C for another 0, 24 or 44 h. ^a, b, c, d^ mean values not sharing the same letters are significantly different, *P* < 0.05 (*n* = 4)

Gene and protein expressions of β-actin and γ-actin were detected at 0 h, 24 h and 44 h of COCs *in vitro* maturation with or without ovarian HS, as shown in Fig. 3. Results showed that the gene and protein expression levels of β-actin and γ-actin from control group CCs increased gradually with the oocyte development, among which both actins’ gene expression and the β-actin protein expression level in CCs were remarkably up-regulated (*P* < 0.05) at 24 h and 44 h of *in vitro* maturation when compared with 0 h. Meanwhile, ovarian HS significantly reduced (*P* < 0.05) the 24 h and 44 h gene expression levels, and the 24 h protein expression levels of both β-actin and γ-actin in CCs cultured *in vitro*, when compared with control group.

### TZPs variation during the oocyte development

**Fig. 4:**
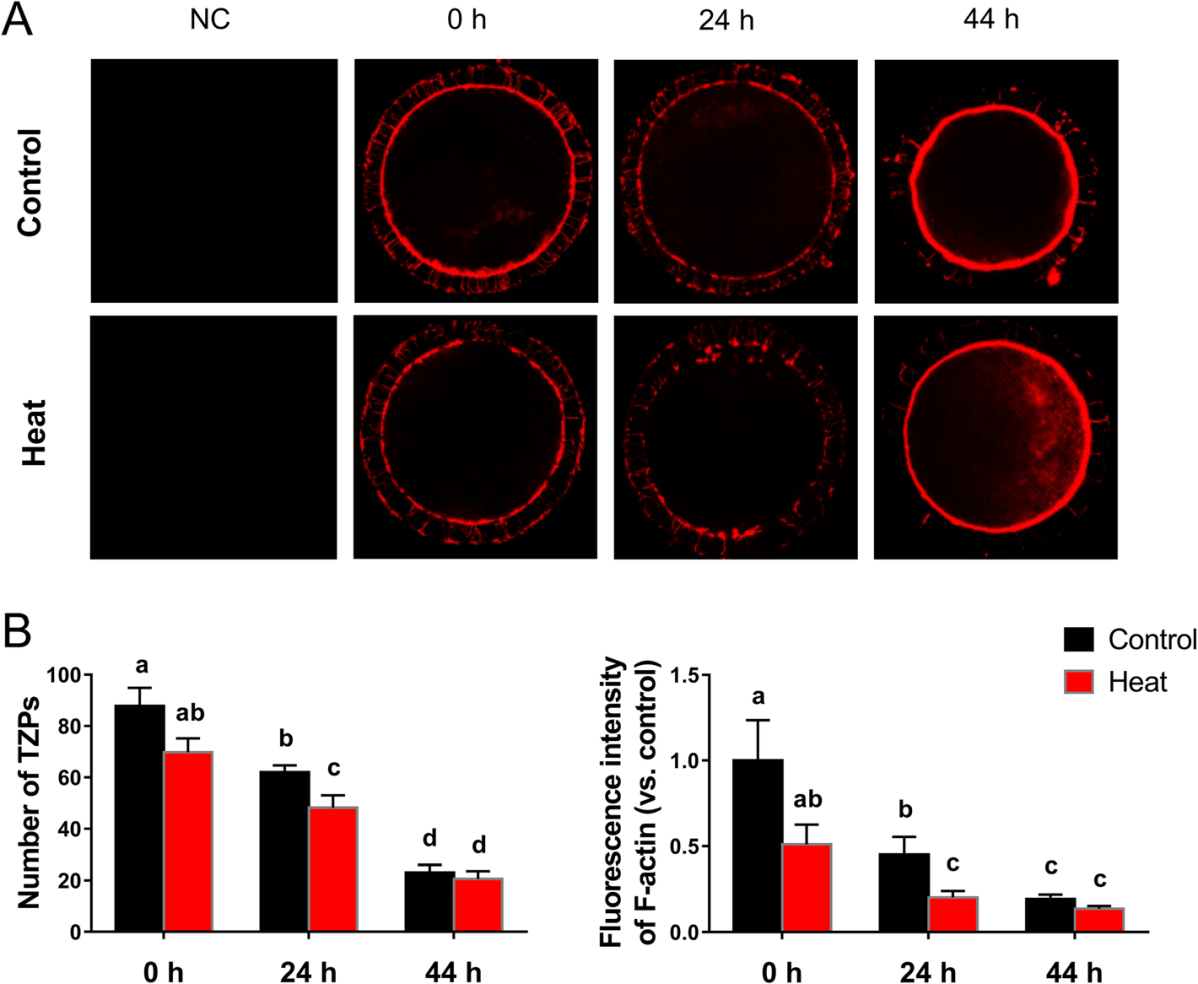
Representative images and the statistics of intercellular TZPs structures after heat exposure. (**a**) Images representing the TZPs variations during the oocytes *in vitro* maturation; (**b**) Changes of TZPs numbers during the oocytes development; (**c**) Changes of fluorescence intensity of F-actin in TZPs structures during the oocytes development. Data are expressed as mean ± SEM. Control, ovaries were kept in 38.5 °C water bath for 1 h, and then the isolated COCs were cultured in 38.5 °C for 0, 24 or 44 h; Heat, ovaries were kept in 41.5 °C water bath for 1 h, and then COCs were isolated and cultured in 38.5 °C for another 0, 24 or 44 h. ^a, b, c, d^ mean values not sharing the same letters are significantly different, *P* < 0.05 (*n* = 30)

The changes of TZPs structures during the oocyte development were identified by staining with the fluorescent dye TRITC Phalloidin (Fig. 4a). Data showed that both the number of TZPs and the fluorescence intensity of F-actin in TZPs decreased gradually during the oocyte *in vitro* maturation. Ovarian HS treatment significantly reduced (*P* < 0.05) the number of TZPs and the fluorescence intensity of F-actin in TZPs at the 24 h stage of COCs *in vitro* maturation, when compared with control groups (Fig. 4b).

### Colocalization analysis of G-actins with F-actin in TZPs

**Fig. 5:**
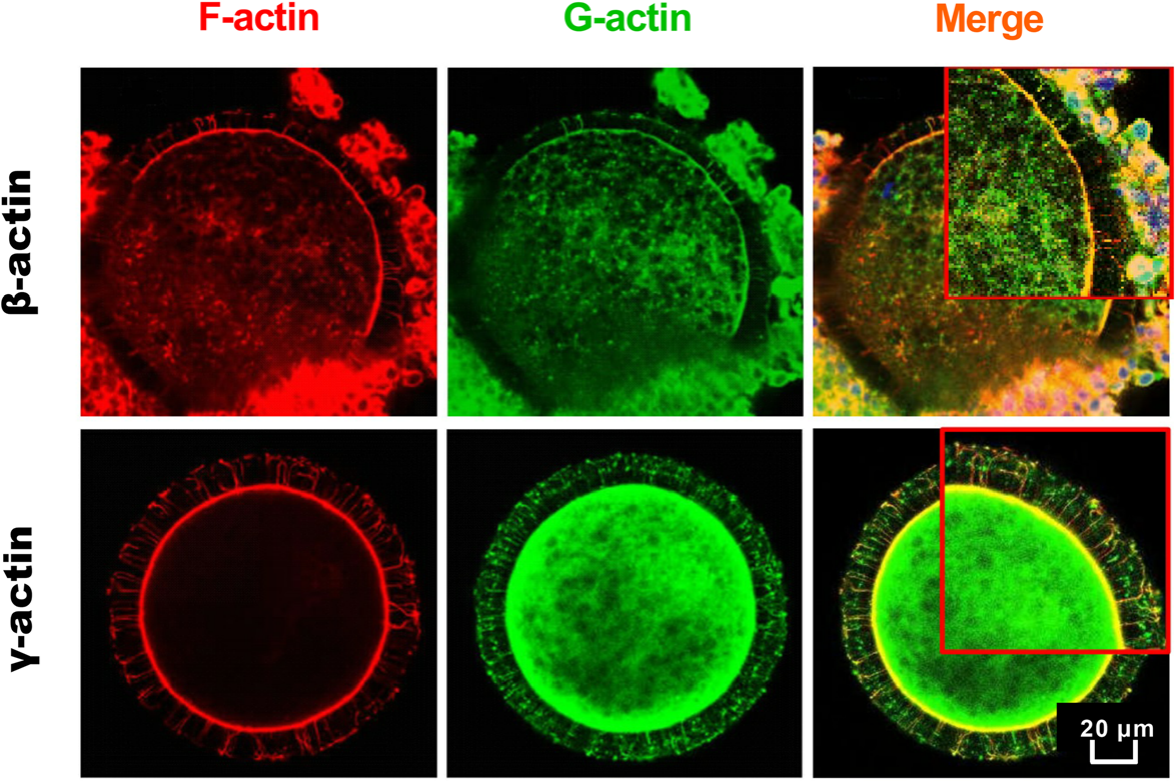
Representative images of the colocalization relationships between F-actin and β-actin or γ-actin. All COCs were cultured in 38.5 °C *in vitro* for 24 h after the ovarian HS

To identify the detailed composition of F-actin in TZP structures, we detected the expression levels of β-actin and γ-actin and their colocalization areas with F-actin in TZPs, by using the fluorescent dye TRITC Phalloidin and anti-actins immunofluorescence (Fig. 5). Results revealed that both β-actin and γ-actin show strong fluorescence signals in the colocalization areas with F-actin, indicating the involvement of these two monomeric actins in the F-actin formation.

### Functions of TZPs and GJs between CCs and oocytes after ovarian HS

**Fig. 6:**
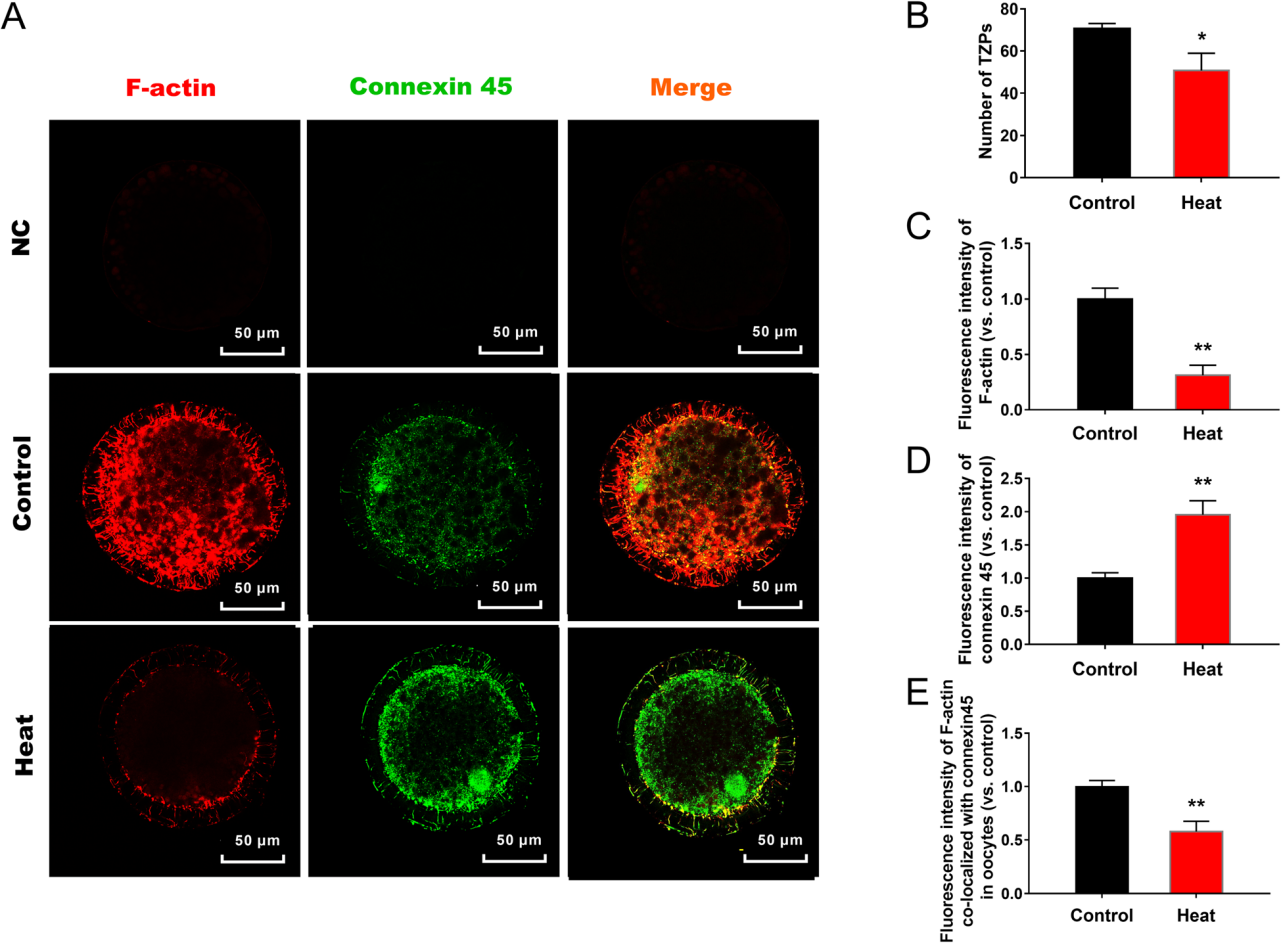
Microscopic imaging analysis of TZPs and GJs in porcine oocytes. **a** Representative staning images of TZPs and GJs structures in oocytes, markered by F-actin and connexin 45, respectively; (**b**-**e**) Statistics of TZPs numbers (**b**) and the fluorescent intensities of TZPs (**c**), GJs (**d**) and their co-staining areas (**e**) in oocytes, markered by F-actin and connexin 45, respectively. Data are expressed as mean ± SEM. Control, ovaries were kept in 38.5 °C water bath for 1 h, and then the isolated COCs were cultured in 38.5 °C for 24 h; Heat, ovaries were kept in 41.5 °C water bath for 1 h, and then COCs were isolated and cultured in 38.5 °C for another 24 h. *P < 0.05 and **P < 0.01, compared with control (*n* = 30)

The changes of TZPs and GJs were detected at the stage of 24 h *in vitro* maturation by using the fluorescent dye TRITC Phalloidin and the anti-connexin 45 immunofluorescence (Fig. 6a). Results showed that though HS significantly (*P* < 0.05) increased the fluorescence intensity of GJs marker protein connexin 45 on oolemma (Fig. 6d), whereas the number of TZPs and the F-actin fluorescence intensity in zona pellucida as well as the co-staining areas of TZPs and GJs on oolemma were remarkably decreased by HS (Fig. 6b, c, e).

## Discussion

The persistent seasonal infertility in domestic animals has been drawing increasing concern of the disruptive effects of HS on oocyte quality and its subsequently embryonic development in pigs [12, 13], cows [10, 14] and mouse [11, 15]. The demonstrated mechanisms refer to the mitochondrial dysfunctions, oxidative stress and cell apoptosis in both oocytes and granulose cells [12, 16–19]. Denuded oocytes or COCs were commonly used in these researches to simulate the microenvironment *in vivo*, due to the advantages of easier accessibility and less variables in culture condition. However, these models are quite distant from the physiological environment, thus the role of ovarian structure and the relationship between oocytes and other follicular factors were always neglected. Therefore, whole porcine ovaries, which have a more anatomically integrated structure than denuded oocytes and COCs, were employed in this study to investigate the responses of CCs and oocytes after HS exposure, as reported by Pennarossa, et al. [23].

The superior quality and high embryonic development potential of oocyte is largely depend on the optimal nuclear and cytoplasmic maturation before ovulation [28]. High quality oocyte is usually characterized by numerous typical features, such as the well organized CCs [29, 30], high-proportioned oocyte survival rate and first polar body extrusion rate [31], favourable pregnancy outcomes [32], and optimal numbers and status of mitochondria [33, 34]. While, mitochondrial dysfunction, which is characterized by abnormal mitochondrial distribution patterns, irregular mtDNA-encoded genes expression and superfluous mROS generation [35, 36], will result in the compromised quality in oocytes. Nevertheless, the effects of HS on oocyte maturation as well as the mitochondrial functions are actually dependent on the duration and the intensity of stresses. For example, porcine COCs treated at 41.5 °C for 1 h *in vitro* significantly enhanced the mitochondrial degradation and biogenesis, and thus improved the development of oocytes into blastocysts [37]. On the contrary, exposing bovine oocytes to repeated HS (40.5 °C, 10 h per day for 2 days) resulted in significant reductions in mitochondrial membrane potentials and obvious impairments in oocyte quality and its blastocyst development [38]. Inconsistent with the previous studies, we found here that ovaries subjected to 41.5 °C for 1 h before the COCs *in vitro* maturation significantly impaired the oocyte quality and the mitochondrial functions in pigs, as revealed by impaired cumulus expansion, delayed meiotic resumption, lower survival rate and first polar body extrusion rate, and remarkably decreased mtDNA-encoded gene expression levels. On one hand, this may be resulted from the concurrent HS effects induced by multiple factors existing in the ovaries [23]. Meanwhile, another difference could be that in our experiment, the oocytes were exposed to HS within the ovarian follicles, when they had not resumed meiosis. While in the previous study, HS was imposed after meiotic resumption, which can be induced by the removal of COCs from the follicles.

Interactions between CCs and oocytes are of great importance in the oocyte maturation. On one hand, CCs provide oocytes with various nutrients and signaling molecules to promote their growth and development [2, 39]. In turn, oocyte regulates the expansion [40], proliferation, differentiation [41] and apoptosis [42] of CCs through the paracrine pathways. TZPs are one class of membrane-specialized structures of CCs, which responsible for the information and material exchanges by contacting with oocytes through the GJ structures distributed on oolemma [1]. Previous report showed that during the oogenesis process, the expression of GJ proteins increase notably in responding to the enlarged volume and growing material demands of oocytes [43]. On the contrast, TZPs are particularly numerous during the pre-antral stages of oocytes, their number and complexity of contact at the oocyte surface decrease gradually during the following developmental periods [8, 44]. Similarly, our previous study also demonstrated that GJs protein was remarkably up-regulated, but TZPs were obviously disrupted in porcine COCs after the long-term HS treatment [20]. In agreement with the previous publication, we found in this study that, although the expression of GJ marker protein connexin 45 on oolemma increased significantly post HS, the number of TZPs structures decreased gradually with the oocyte development. Moreover, we noticed that HS merely reduced the TZPs number and its fluorescence intensity in the areas colocalized with GJs at 24 h of COCs *in vitro* maturation. This finding implicate that the dysfunctions of CCs induced by HS, especially at the first 24 h of *in vitro* maturation, may contribute to the delayed maturation of oocytes that have the highest sensitivity to the external stimuli during this period [13]. Nevertheless, further evidences are still needed to clarify the underlying mechanisms of TZPs abnormalities under HS.

F-actin is the main component of TZPs [5], which is formed by the monomeric β-actin and γ-actin [6, 7] through activation, polymerization and space torsion [45, 46]. Reports from drosophila and rabbit showed that hyperthermia stimulations during the embryonic development *in vitro* would result in the destabilization of F-actin, which subsequently increases the chance of morphogenesis mistakes and causes apoptosis in embryos [47, 48]. Studies about other somatic cells also demonstrated the strong correlations between cell functions and the F-actin reassembly or actin gene expressions under HS [21, 22]. These researches, from another perspective, expanded our understandings about the possible mechanisms of F-actin reorganization and G-actin expression in leading to the TZPs abnormalities under HS. In our study, both β-actin and γ-actin produced by CCs were demonstrated to played critical roles in constituting the TZPs structures in oocytes. The fluorescence intensity of F-actin and the number of TZPs in zona pellucida decreased gradually, whereas the expression of β-actin and γ-actin from control group CCs increased gradually with the oocyte development, both from transcriptional and translational levels, suggesting that though more and more monomeric actins were synthesized in CCs during the oocyte maturation, less of them were assigned to construct the TZP structures. Furthermore, HS aggravated the TZPs disruptions by decreasing the G-actins mRNA and protein abundances at 24 h and (or) 44 h stages of COCs *in vitro* maturation. The results hint us that reduced actin expression efficiency in CCs is strongly associated with the TZPs disruptions in COCs, which can subsequently contribute to the compromised quality of oocytes under HS.

## Conclusions

The present study showed us that the immature oocytes in pigs have the most numerous TZPs structures, while their number and contact with oocyte decreased gradually during the following developmental processes. Both β-actin and γ-actin were shown to play critical roles in constituting the TZPs structures and promoting the oocyte maturation, while ovarian HS significantly reduced the TZPs structure formation through the down-regulation of monomeric actin expressions in CCs, from both transcriptional and translational levels. These results indicate that decreased actin expressions in CCs induced by HS is strongly associated with the TZPs disruptions, which can subsequently result in the compromised oocyte quality and the embryonic development reduction during the female reproductions.

## Data Availability

The datasets used and/or analysed during the current study are available from the corresponding author on reasonable request.

## Abbreviations

TZPs: Transzonal projections
CCs: Cumulus cells
HS: Heat stress
COCs: Cumulus-oocyte complexes
F-actin: Filamentous actin
G-actin: Monomeric actin
GJ: Gap junction
CCD: Charge coupled device camera
TCM-199: Tissue culture medium 199
mtDNA: Mitochondrial DNA
mROS: Mitochondrial reactive oxygen species
IF: Immunofluorescence
